# Drawing parallels between amyloid PET in Alzheimer’s disease and amino acid PET in primary and secondary brain tumors

**DOI:** 10.1093/noajnl/vdag043

**Published:** 2026-02-16

**Authors:** Nelleke Tolboom, Antoine Verger, Matthias Preusser, Marjolein Geurts, Philip Scheltens, Nathalie Lisa Albert

**Affiliations:** Department of Radiology and Nuclear Medicine, University Medical Centre Utrecht, Utrecht, The Netherlands (N.T.); Department of Nuclear Medicine and Nancyclotep Imaging Platform, IADI, INSERM U1254, Université de Lorraine, Nancy, France (A.V.); Division of Oncology, Department of Medicine I, Medical University of Vienna, Vienna, Austria (M.P.); Department of Neurology, Brain Tumor Center at Erasmus MC Cancer Institute, Rotterdam, The Netherlands (M.G.); Department of Medical Oncology, Erasmus MC Cancer Institute, Rotterdam, The Netherlands (M.G.); EQT Life Sciences Amsterdam Amsterdam, The Netherlands (P.S.); of Nuclear Medicine, LMU University Hospital, LMU LMU Munich, Munich, Germany (N.A.)

## Abstract

The evolution of amyloid PET in Alzheimer’s disease illustrates the potential path of amino acid PET in neuro-oncology. Initially seen as of limited value, amyloid PET ultimately enhanced diagnostic accuracy, guided management, and became central once therapies demonstrated PET-measured efficacy. Amino acid PET for CNS tumors is at a similar turning point. It refines diagnosis, distinguishes progression from treatment effects, and supports treatment planning. As demand grows and tracer access improves, amino acid PET could follow amyloid PET’s trajectory: emerging as a key tool in precision medicine and clinical management and as a surrogate endpoint in therapeutic trials.

In the rapidly evolving field of neuroimaging, the journey of added clinical and diagnostic value demonstrated by amyloid PET in Alzheimer’s disease offers key insights into the potential trajectory of amino acid PET in neuro-oncology. Early clinical adoption of amyloid PET was limited by concerns regarding the absence of clear therapeutic implications. However, research showed that amyloid PET clearly improved diagnostic accuracy, greatly enhanced physician’s confidence in the diagnosis, predicted cognitive decline, changed management and showed that an accurate diagnosis with PET could even delay nursing home placement and reduced health care costs.^1^^,^^2^

The biggest change for amyloid PET occurred when monoclonal antibodies targeting amyloid could significantly lower amyloid burden on PET, to the extent that the visual read became negative after 12-18 months.^3^^,^^4^ This led to the initial first accelerated approval of aducanumab in June 2021, as the U.S. Food and Drug Administration (FDA) reasoned that this effect on the biomarker would translate into a clinical benefit later on, as such providing a surrogate endpoint for assessing treatment efficacy. Continued approval was contingent on verifying clinical benefit in confirmatory trials, which were ultimately unsuccessful and led to the discontinuation of aducanumab. However, since then, two other monoclonal antibodies (lecanemab and donanemab) have been approved, based on the combined effect on PET and clinical efficacy. As appropriate use recommendations mention either amyloid PET or cerebrospinal fluid (csf) studies indicative of Alzheimer’s disease for selection of patients for treatment (lecanemab and donanemab) and amyloid PET validated to assess amyloid plaque clearance in response to treatment (donanemab), this has led to the use of amyloid PET in regular patient care to select and monitor patients. Amyloid PET thus gained momentum after demonstrating therapeutic impact with monoclonal antibodies. Rapid adoption of amyloid PET was further driven by strong industry engagement, including the development of commercially available tracers, and sponsorship of large-scale collaborative initiatives to advance biomarker-based trial enrichment, notably the Alzheimer’s Disease Neuroimaging Initiative. Real clinical adoption in the United States followed Medicare’s decision to reimburse PET scans as part of the treatment workup. Its value is rooted in its ability to visualize early effects of pathology, well before irreversible structural damage occurs, thereby enabling an earlier accurate diagnosis and further our understanding of Alzheimer’s disease.

## Amino Acid PET Is Now at a Similar Point in Time as Amyloid PET Once Was

Like amyloid PET, amino acid PET offers insight into a biological process. In contrast to Alzheimer’s disease, the PET ligand does not target the same molecular substrate as the current therapy but reflects amino acid metabolism of the tumor. Notably, an experimental radionuclide-based therapy targeting the same mechanism is currently under investigation.^5^

Even though MRI addresses a broader range of clinically relevant questions in CNS tumors compared with Alzheimer’s disease, the specific aspect of amino acid metabolism cannot be visualized with standard MRI. PET therefore provides complementary information to MRI because tracer uptake occurs independently of blood–brain/blood-tumor barrier disruption^6^ and helps to differentiate between treatment-induced changes and tumor recurrence in cases with equivocal MRI.^7^

While like in Alzheimer’s disease amino acid PET has value at the initial differential diagnosis, it also has additional clinical value along the whole diagnostic trajectory including the follow-up phase.^7^ However, unlike in Alzheimer’s disease, where amyloid and related measurements in csf and blood-based biomarkers have demonstrated clinical value, alternative biomarkers have yet to prove their usefulness in neuro-oncology. Several studies and multicenter evaluations have demonstrated clinical value of amino acid PET for patients with primary brain tumors and brain metastases:^7-9^ amino acid PET significantly enhances the ability to distinguish true tumor progression from treatment-related effects, provides diagnostic accuracy and potentially alters clinical management. Emerging evidence suggests amino acid PET uptake metrics might have prognostic value in IDH-mutant glioma and may help indicate early temozolomide response. Beyond this, amino acid PET may contribute to improved treatment planning by enabling more precise delineation of tumor extent, including regions that are metabolically active but not contrast-enhancing on MRI, although clinical benefit remains to be shown. These advantages have been recognized in the European Association for Neuro-Oncology (EANO) guidelines,^7^^,^^10^ which include amino acid PET as a potential diagnostic tool in specific clinical scenarios.

## Widespread Clinical Adoption of Amino Acid PET Has Been Slow Despite Increasing Research Efforts

Regardless of evidence supporting the clinical value of [^11^C] methionine PET in gliomas since 1983,^11^ adoption of amino acid PET was slow due to the short half-life of ^11^C, restricting use to centres with on-site cyclotrons. As observed with the transition from [^11^C]PIB to ^18^F labelled amyloid tracers, broader availability was achieved following the subsequent development of ^18^F labelled amino acid tracers. Despite these advances, the world-wide commercial availability of amino acid PET tracers is currently largely limited to Europe^12^ due to regulatory and practical constraints. However, this situation may change in the near future, as resubmission of a New Drug Application for [^18^F]FET to the U.S. Food and Drug Administration (FDA) is anticipated. In sites where amino acid PET is commercially available, many neuro-oncologists cite the absence of level 1 evidence as a barrier to routine use.^7^ Added to this is the persistent perception that PET is expensive, which overlooks data showing favorable cost-effectiveness and the potential for PET to reduce unnecessary treatments and follow-up procedures.

The ongoing adoption of PET-based Response Assessment in Neuro-Oncology criteria,^13-15^ alongside the development of novel therapies targeting specific metabolic pathways in glioma and brain metastases, underscores the growing importance of amino acid PET. Beyond resolving diagnostic uncertainties, amino acid PET has the potential to serve as a surrogate endpoint in clinical trials, just as amyloid PET has done in Alzheimer’s. Central driver for clinical adoption is the introduction of novel, high-cost therapies associated with risks of adverse effects such as IDH inhibitors, immunotherapies, antibody–drug conjugates for primary and secondary brain tumors. Demonstrating that PET can improve therapeutic precision by identifying patients most likely to benefit,^16^^,^^17^ while avoiding ineffective or potentially harmful treatment in non-responders, is essential to addressing reimbursement concerns. This also highlights the importance of generating strong evidence through well-designed prospective trials.

The new criteria therefore represent a promising step forward in precision oncology and drug development. While they are not yet validated surrogate endpoints, their early integration into exploratory trials and regulatory flexibility for unmet medical needs could significantly impact both clinical practice and approval processes, provided further evidence and consensus are built.

Although incidence and prevalence of dementia are not comparable with those of CNS tumors and the accompanying potential market for Amyloid PET is larger than the current market for amino acid PET, the clinical potential of amino acid PET in CNS tumors is likely broader. In CNS oncology, PET can provide clinically relevant information at multiple stages of the disease course.^7^^,^^14^ Consequently, individual patients may undergo multiple PET examinations over the course of their disease, mirroring established PET use in extracranial oncology. In contrast, amyloid PET has limited indications for repeated imaging in routine care. It is important to note that, although gliomas represent the most extensively studied and clinically validated indication for amino acid PET to date, brain metastases, as they are by far the most common intracranial tumors in adults, are likely to constitute the largest future clinical and market application.

## The Experience of Amyloid PET Demonstrates that Amino Acid PET Can Become a Cornerstone of Clinical Practice When its True Value is Recognized

Embracing amino acid PET is not just about adding another diagnostic tool, it is about working towards a highly needed tool that ensures patients with glioma and brain metastases receive the right treatment at the right time and that clinical trials enroll patients most likely to benefit. To disregard this opportunity would be to delay the path to advanced patient care.
